# Supplementary material for: A molecular view on the escape of lipoplexed DNA from the endosome
Source: eLife. 2020 Apr 16;9:e52012. doi: 10.7554/eLife.52012 (PMC7170654; doi:10.7554/eLife.52012)
Supplement: Supplementary file 1. [file elife-52012-supp1.docx]

**DOTAP validation.**

*The bilayer area per lipid (APL) and bilayer thickness (BT) were compared to experiments and AA simulations.* *The DOTAP parameters can be found online at* ‘<http://cgmartini.nl/index.php/force-field-parameters/lipids2/350-lipid-details>’.

|  | **Property** | **Mean** | **SEM** |
| --- | --- | --- | --- |
| **CG** | APL(nm^2^) | .70* | 4.14E-05 |
| **AA^**^** | APL(nm^2^) | .64 | 1.00E-02 |
| **CG** | BT(nm) | .38* | 4.15E-04 |
| **AA^**^** | BT(nm) | .39 | 5.00E-02 |
| **Experimental^***^** | BT(nm) | .37 | 2.00E-01 |

*The rounding error was larger than the SEM.

** [Pokorna, S., Jurkiewicz, P., Cwiklik, L., Vazdar, M. & Hof, M. Interactions of monovalent salts with cationic lipid bilayers. *Faraday Discuss.* **160**, 341–58; discussion 389–403 (2013).](http://paperpile.com/b/Wdcx4d/0W9At)

*** [Leonenko, Z. V. & Cramb, D. T. Revisiting lipid – general anesthetic interactions (I): Thinned domain formation in supported planar bilayers induced by halothane and ethanol. *Can. J. Chem* **82**, 1128–1138 (2004).](http://paperpile.com/b/Wdcx4d/c8IFe)
