# Supplementary material for: A molecular view on the escape of lipoplexed DNA from the endosome
Source: eLife. 2020 Apr 16;9:e52012. doi: 10.7554/eLife.52012 (PMC7170654; doi:10.7554/eLife.52012)
Supplement: Supplementary file 2. [file elife-52012-supp2.docx]

**Lipoplex membrane mixture properties.**

*The APL and bilayer thickness were compared between the different lipoplex formulations. The tested compositions were a 1:4 ratio of either DPTAP, DOTAP, DLiTAP with DOPE. The XXTAP variant is used to indicate these mixtures and their properties.*

|  | **Property** | **Mean** | **SEM** |
| --- | --- | --- | --- |
| **DPTAP** | APL(nm^2^) | .63 | 4.28E-5 |
| **DOTAP** | APL (nm^2^) | .63 | 2.31E-4 |
| **DLiTAP*** | APL (nm^2^) | .66 | 8.37E-5 |

*Differs 5% from the DPTAP and DOTAP in APL
